# Effectiveness of Aspirin Plus Dipyridamole Versus Clopidogrel in Preventing Recurrent Ischemic Stroke: A Systematic Review

**DOI:** 10.7759/cureus.93790

**Published:** 2025-10-03

**Authors:** Agbonmwanre E Osayagbon, Aliyu O Olaniyi, Japheth Oyovwi, Anup Banerjee, Olabisi P Lawal, Muhammad A Butt

**Affiliations:** 1 Geriatrics, Stepping Hill Hospital, Manchester, GBR; 2 General Medicine, Stepping Hill Hospital, Manchester, GBR; 3 Acute Internal Medicine, Stepping Hill Hospital, Manchester, GBR; 4 Medical Laboratory Science, University of Benin, Benin City, NGA; 5 Medicine, Stepping Hill Hospital, Manchester, GBR

**Keywords:** aspirin, clopidogrel, dipyridamole, ischemic stroke, secondary prevention

## Abstract

This systematic review evaluated the comparative effectiveness of aspirin plus extended-release dipyridamole (Asp+Dp) versus clopidogrel in preventing recurrent ischemic stroke. Three eligible studies were included, enrolling a total of 21,752 participants with a cumulative follow-up of 32 months. Many participants were recruited from the large-scale PROFESS trial (20,332 participants). Intervention dosing consisted of aspirin 25 mg combined with extended-release dipyridamole 200 mg administered twice daily, while the comparator was clopidogrel 75 mg once daily. In the PROFESS trial, recurrent stroke occurred in 916 patients (9.0%) in the Asp+Dp group and 898 patients (8.8%) in the clopidogrel group. The combined risk of recurrent stroke or major haemorrhagic events was comparable between groups, affecting 1,194 participants (11.7%) receiving Asp+Dp and 1,156 participants (11.4%) receiving clopidogrel. With respect to antiplatelet activity, one randomised pilot study demonstrated that Asp+Dp was associated with a delayed but significant reduction in the expression of several activation-dependent platelet receptors. Conversely, clopidogrel monotherapy was associated with earlier and more potent antiplatelet activity. Clinically, these study findings suggest that clopidogrel exerts more potent and earlier antiplatelet effects, whereas Asp+Dp produces broader but delayed downregulation of multiple platelet activation pathways. Regarding functional outcome recurrence, mortality, bleeding risk, or serious adverse events in patients with acute, mild ischemic stroke, no significant differences were observed between Asp+Dp and clopidogrel, with both regimens being practical and feasible for clinical use. In conclusion, while both therapies demonstrate comparable clinical effectiveness, further research is warranted to evaluate their long-term impact on quality of life in patients with ischemic stroke.

## Introduction and background

Stroke, or cerebrovascular accident, is one of the leading causes of death and long-term disability worldwide, with significant clinical, social, and economic impacts [1]. Globally, an estimated 10 million people experience a first stroke each year, and more than 6.5 million die from its consequences [1]. Stroke is also the primary cause of adult-acquired disability, leaving survivors with varying degrees of functional impairment [2]. The burden is substantial in both developed and developing nations, with low- and middle-income countries facing a rising incidence due to ageing populations and lifestyle changes [3].

Approximately 80% of strokes are ischemic, caused by obstruction of cerebral blood flow, and survivors face a considerable risk of recurrence [4]. Recurrent ischemic stroke accounts for a large proportion of stroke-related morbidity, mortality, and healthcare costs [5]. In the United Kingdom, around 30,000 people experience a recurrent stroke annually, with most recurrences occurring within the first year after the initial event [6]. Preventing recurrence is therefore a central focus of secondary prevention strategies, which include risk factor modification (hypertension, diabetes, hyperlipidaemia, lifestyle) plus antiplatelet therapy.

Antiplatelet therapy is a cornerstone of secondary prevention in patients without a cardioembolic source of stroke [7]. Several regimens are available, most notably aspirin, clopidogrel, and the combination of aspirin with extended-release dipyridamole (Asp+Dp) [7,8]. Mechanistically, clopidogrel led to faster inhibition of platelet aggregation through the P2Y12 receptor pathway, whereas aspirin-dipyridamole targeted alternative platelet activation pathways. Clinical guidelines from the American Heart Association/American Stroke Association recommend these agents as first-line options for the secondary prevention of ischemic stroke [9]. However, despite widespread use, uncertainty persists regarding whether Asp+Dp offers superior protection compared with clopidogrel monotherapy [10].

A recent systematic review and meta-analysis by Hilkens et al. (2021) evaluated the benefits and risks of clopidogrel versus aspirin monotherapy after ischemic stroke, providing updated evidence on the comparative effectiveness of these antiplatelet regimens for secondary prevention [10]. Also, the MATCH trial (Diener et al., 2004) compared aspirin plus clopidogrel with clopidogrel alone in high-risk patients after recent ischemic stroke or transient ischemic attack (TIA), finding no significant reduction in recurrent stroke but an increased risk of major bleeding with combination therapy [11]. In contrast, the PROFESS trial directly compared Asp+Dp with clopidogrel but found no clear difference in overall stroke recurrence rates [12]. More recent analyses have continued to report conflicting evidence, with some data suggesting differences in platelet inhibition profiles but no consistent superiority in clinical outcomes [13].

This review specifically focuses on Asp+Dp and clopidogrel, rather than dual antiplatelet therapy (e.g., aspirin + clopidogrel) or newer agents, such as ticagrelor, because these two regimens are the most established, widely recommended first-line options in international guidelines and continue to be the subject of clinical debate. Clarifying their comparative effectiveness is directly relevant to everyday clinical decision-making. Hence, this uncertainty presents a significant gap in clinical decision-making. Physicians must weigh potential differences in efficacy and side-effect profiles, such as headache associated with dipyridamole, and practical considerations like cost and dosing frequency [14,15]. Given the substantial number of patients worldwide who require long-term secondary prevention, clarifying the comparative effectiveness of these regimens has important implications for patient care and healthcare policy.

Objective and research question

The objective of this structured literature review was to synthesize current evidence from randomized controlled trials (RCTs) and high-quality pilot studies comparing the effectiveness of Asp+Dp versus clopidogrel monotherapy in the secondary prevention of ischemic stroke. Specifically, the review aimed to address the following research question: is aspirin plus dipyridamole more effective than clopidogrel alone in preventing recurrent ischemic stroke and improving functional outcomes in adult patients?. This review appraises recent evidence to inform clinical practice, guide prescribing, and identify research gaps.

## Review

Materials and methods

Study Design

This study was a structured literature review conducted in accordance with Preferred Reporting Items for Systematic Reviews and Meta-Analyses (PRISMA) guidelines [16]. The review sought to identify and synthesise evidence from randomised controlled trials (RCTs) and high-quality pilot studies comparing Asp+Dp with clopidogrel monotherapy in the secondary prevention of ischemic stroke.

Data Sources and Search Strategy

A comprehensive literature search was performed in two electronic databases: MEDLINE (via PubMed) and CINAHL [17,18]. The search covered the period from January 2008 to January 2025 and was limited to studies published in English. Search terms were developed using the PICO (Population, Intervention, Comparison, Outcome) framework [19] and combined using Boolean operators. Keywords included: Population - “stroke recurrence,” “transient ischemic attack,” “cerebrovascular accident”; Intervention: “aspirin,” “dipyridamole,” “aspirin plus dipyridamole”; Comparison - “clopidogrel”; Outcome - “secondary prevention,” “recurrent stroke,” “functional outcome,” “quality of life”. Table 1 shows the PICO framework for the study.

**Table 1:** PICO framework PICO: Population, Intervention, Comparison, Outcome

Component	Description
Population (P)	Adults with prior ischemic stroke or transient ischemic attack (TIA)
Intervention (I)	Aspirin 25 mg + extended-release dipyridamole 200 mg twice daily
Comparison (C)	Clopidogrel 75 mg once daily
Outcomes (O)	Primary: recurrent ischemic stroke; Secondary: functional outcomes (modified Rankin Scale, quality of life), composite vascular events, adverse events (bleeding, headache)
Study Design	Randomized controlled trials (RCTs) and a pilot study (total n=21,752)

Database-specific indexing terms (e.g., MeSH in MEDLINE) were applied where appropriate [20]. Boolean operators “OR” were used to combine synonyms, and “AND” was used to link PICO components. Wildcards and truncations were employed to capture variations in spelling [21].

Inclusion and Exclusion Criteria

The inclusion criteria for this review were studies that involved adults with a history of ischemic stroke or transient ischemic attack (TIA). Eligible studies assessed aspirin in combination with extended-release dipyridamole as the intervention, compared with clopidogrel monotherapy. Outcomes of interest included recurrent ischemic stroke and functional outcomes such as the modified Rankin Scale and quality-of-life measures. Only RCTs and pilot studies were considered.

Exclusion criteria were applied to studies that involved populations with hemorrhagic stroke, assessed other antiplatelet regimens, such as ticagrelor or triple therapy, or employed observational or qualitative study designs. Additionally, studies published in languages other than English and those published before 2008 were excluded.

Study Selection

All identified citations were imported into reference management software (RefWorks) for duplicate removal [22]. Titles and abstracts were screened against inclusion criteria, and full texts were retrieved for potentially eligible studies. A second round of screening was conducted on the full texts to confirm inclusion. Discrepancies were resolved through discussion.

Data Extraction

Data were extracted using a standardized form adapted from the Research Council for Complementary Medicine (RCCM) template, which includes sections for bibliographic information, study characteristics, participant details, interventions, comparators, outcomes, quality assessment, and reviewer observations [23]. Extraction was conducted independently by two reviewers to ensure consistency and minimize bias. Collected data included study characteristics (authors, year, country, design), intervention and comparator details, outcome measures, and follow-up duration. Discrepancies were resolved through discussion, with a third senior reviewer adjudicating when consensus could not be reached, ensuring accuracy, completeness, and reproducibility of the data.

Quality Assessment

The methodological quality of included studies was assessed using the Cochrane Collaboration’s Risk of Bias Tool [24]. The following domains were evaluated: random sequence generation, allocation concealment, blinding of participants and personnel, blinding of outcome assessors, incomplete outcome data, selective outcome reporting, and other sources of bias.

Data Synthesis

Given the small number of eligible studies and heterogeneity in outcome measures, a narrative synthesis was performed rather than a meta-analysis [25]. Results were presented descriptively, with relevant statistics (e.g., hazard ratios, odds ratios, confidence intervals, p-values) reported as provided in the original studies.

Study Selection

**Figure 1:**
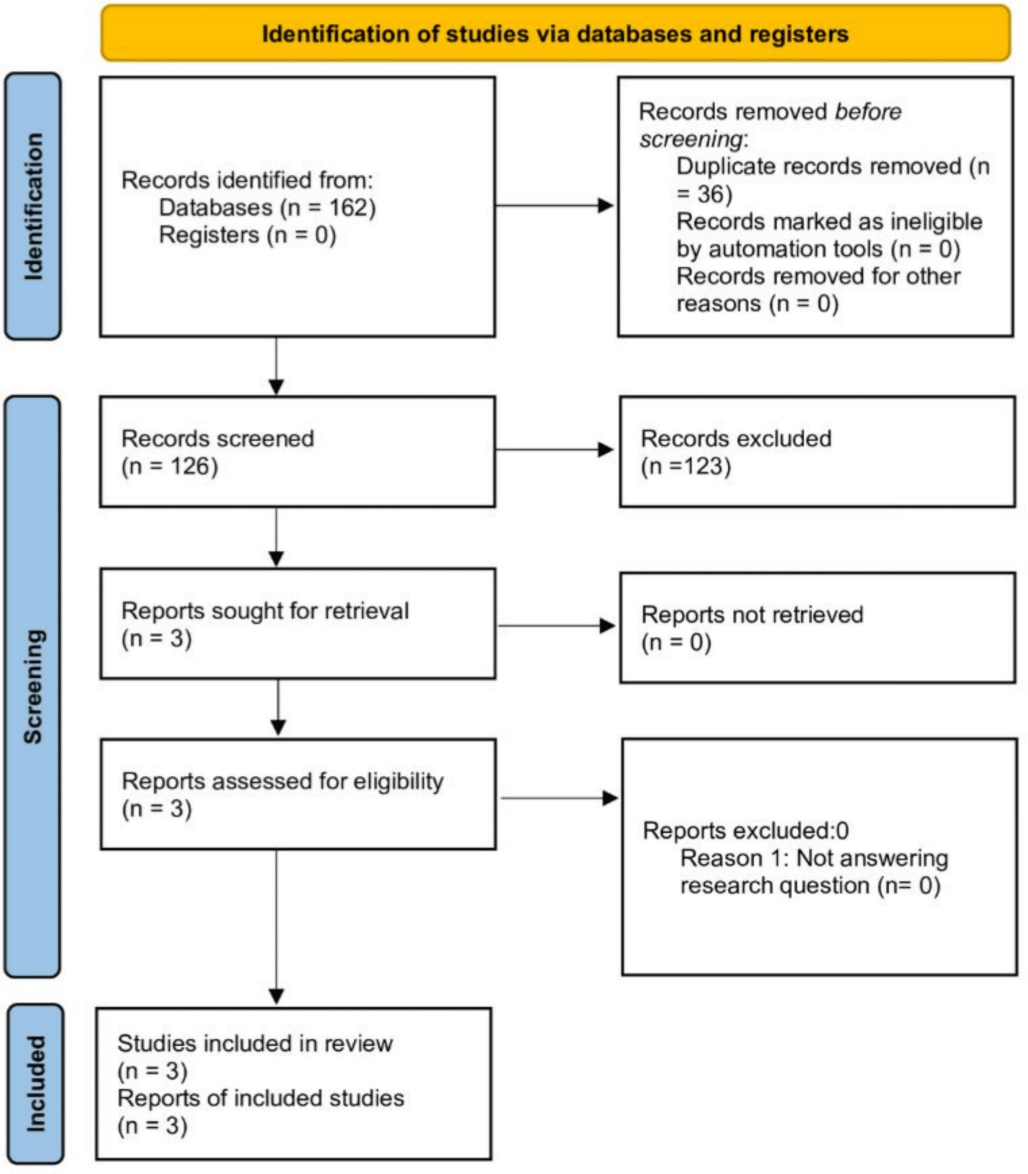
PRISMA 2020 flow diagram for the study selection process Flow diagram illustrating the process of identifying, screening, assessing eligibility, and including studies in the systematic review. The initial search retrieved 162 records in total, 125 from MEDLINE and 37 from CINNAHL. After removing duplicates (n of 36), 126 records remained. One hundred twenty-three articles were irrelevant to the study and were excluded, leaving three studies in the final synthesis: two randomized controlled trials and one pilot study. PRISMA: Preferred Reporting Items for Systematic Reviews and Meta-Analyses

The initial database search identified 162 records (125 from MEDLINE and 37 from CINAHL). After removing duplicates and applying language and date limits, 126 articles remained. Screening by title and abstract excluded studies that did not meet the inclusion criteria. Following a detailed eligibility assessment, three studies met the inclusion criteria and were included in the synthesis: Two RCTs and one pilot study [12,26,27]. The study selection process is shown in the PRISMA diagram in Figure 1.

Study Characteristics

The three included studies, published between 2008 and 2010, were conducted in diverse geographic settings and involved a combined total of 21,752 participants. Two were large multicenter RCTs [12,27] and one was a single-center pilot study [26]. Sample sizes ranged from 60 in the pilot study to 20,332 in the largest RCT. Participant characteristics included adults with prior ischemic stroke or TIA. Mean ages ranged from 60 to 67 years; most participants were male and of European ethnicity. Interventions and comparators were consistent across studies: aspirin 25 mg plus extended-release dipyridamole 200 mg twice daily versus clopidogrel 75 mg once daily. The follow-up duration varied: 30 days, 90 days, and 2.5 years [12,26,27]. The characteristics of the included studies is shown in Table 2. Also, the keywords and number of hits from the CINAHL and MEDLINE databases are shown in Table 3. The pharmacological profile is shown in Table 4.

**Table 2:** Characteristics of included studies Data adapted from Sacco et al. 2008 [12], Serebruany et al. 2008 [26], and Bath et al. 2010 [27].

Author (year)	Country	Study design	Population	Sample size	Intervention	Comparator	Follow-up duration
Sacco et al. (2008) [12]	Multinational	Randomised double-blind active control trial	Recent Ischaemic stroke	20332	Aspirin + dipyridamole	Clopidogrel	2.5 years
Serebruany et al. 2008 [26]	Multinational	Randomised single blind pilot study	Patient aged 40 with a diagnosis of type 2 Diabetes and a history of transient ischaemic attack	60	Aspirin + dipyridamole	Clopidogrel	30 days
Bath et al. 2010 [27]	Multinational	Randomised single blind control trial	Recent ischaemic stroke	1360	Aspirin + dipyridamole	Clopidogrel	90 days

**Table 3:** Keywords and number of hits from the CINAHL and MEDLINE databases

Keywords	Searches	Number of hits (CINAHL)	Number of hits (MEDLINE)
Effectiveness	S1	214346	739610
Efficacy	S2	287101	1267326
Potency	S3	7208	417384
Aspirin	S4	18833	72628
Dipyridamole	S5	1250	10428
Clopidogrel	S6	5352	16286
“Stroke recurrence”	S7	860	2699
“Transient ischaemic attack”	S8	3874	16453
“Cerebrovascular accident”	S9	66832	142138
“Stroke prevention”	S10	12097	10312
S1OR S2 OR S3	S11	479421	2271786
S4 AND S5	S12	403	2285
S7 OR S8 OR S9 OR S10	S13	73235	159114
S12 AND S6	S14	173	497
S11 AND S13 AND S14	S15	37	125

**Table 4:** Pharmacological profile Data adapted from Diener et al. (2004) [11] and Sacco et al. (2008) [12].

Feature	Clopidogrel	Aspirin+ ER- Dipyridamole
Drug Class	Antiplatelet (thienopyridine, P2Y₁₂ inhibitor)	Dual-pathway antiplatelet therapy
Primary Target	Irreversible blockade of platelet ADP P2Y₁₂ receptors	Both COX-1 (aspirin) and PDE/adenosine uptake (dipyridamole)
Mechanism of Action	Prodrug → activated by CYP450 (esp. CYP2C19) → irreversibly inhibits P2Y₁₂ receptor → ↓ ADP-mediated activation of GPIIb/IIIa → ↓ platelet aggregation	Aspirin: ↓ TXA₂ pathway • Dipyridamole: ↑ cAMP/cGMP via PDE inhibition & adenosine uptake blockade → ↓ platelet aggregation & vasodilation
Onset of Action	Detectable within 2 hours; maximal effect after 3–7 days (or faster with loading dose)	Rapid (aspirin) + sustained (ER-DP)
Duration of Action	Irreversible, lasts 7–10 days (platelet lifespan)	Long-lasting due to aspirin’s irreversible effect + dipyridamole’s sustained release
Additional Effects	None (purely antiplatelet)	Vasodilation and improved cerebral blood flow (dipyridamole)
Clinical Effect	Reduces risk of stroke, MI, stent thrombosis, ACS events	Superior secondary prevention of ischemic stroke vs aspirin alone (e.g., ESPS-2, ESPRIT)
Variability in Response	Significant – CYP2C19 polymorphisms affect activation	Less influenced by genetic variability compared to clopidogrel

Risk of Bias Assessment

All studies were rated at a low risk of bias across major domains using the Cochrane Risk of Bias Tool [24]. Randomization and allocation concealment were clearly described in all trials. Two RCTs used double-blinding, while the pilot study used single-blinding. Incomplete outcome data were addressed in all studies, and no evidence of selective outcome reporting was found. The risk of bias assessment (Cochrane RoB domains) is shown in Table 5.

**Table 5:** Risk of bias assessment (Cochrane RoB domain)

Study (year)	Random Sequence Generation	Allocation Concealment	Blinding of Participants/Personnel	Blinding of Outcome Assessment	Incomplete Outcome Data	Selective Reporting	Other Bias	Overall Risk
Sacco et al., 2008 [12]	Low risk	Low risk	Double-blind (Low risk)	Low risk	Low risk	Low risk	None reported	Low
Serebruany et al., 2008 [26]	Low risk	Low risk	Single-blind (Low risk)	Low risk	Low risk	Low risk	None reported	Low
Bath et al., 2010 [27]	Low risk	Low risk	Single-blind (Low risk)	Low risk	Low risk	Low risk	Small sample size	Low-Moderate

Primary Outcomes: Stroke Recurrence

Table 6 shows the outcomes of the included studies.

**Table 6:** Outcomes of included studies Data adapted from Sacco et al. 2008 [12], Serebruany et al. 2008 [26], and Bath et al. 2010 [27].

Author (year)	Primary outcome	Aspirin + dipyridamole	Clopidogrel	P value	Notable adverse event
Sacco et al. 2008 [12]	Recurrent stroke	9.0%	8.8%	P=0.78	Higher incidence of headache in combination
Serebruany et al. 2008 [26]	Change in platelet receptor expression	Moderate delayed antiplatelet activity	Early and more potent antiplatelet activity	-	Headache, transient nausea, and vomiting
Bath et al. 2010 [27]	Functional outcome and recurrence in acute mild ischaemic stroke at day 30 and day 90	672	688	P=0.33	Higher bleeding risk with the combination

Results

The results of our study were as follows.

Sacco et al. (2008) [12]: Their double-blind 2-by-2 factorial trial evaluated 20,332 participants over a mean follow-up of 2.5 years [12]. Recurrent stroke as the primary outcome occurred in 9.0% of patients receiving Asp+Dp and 8.8% of those receiving clopidogrel, with no significant difference between groups (HR = 1.01; 95% CI 0.92- 1.11). Similarly, the composite endpoint of stroke, myocardial infarction, or vascular death (secondary outcome) was observed in 13.1% of participants in both treatment arms (HR = 0.99; 95% CI 0.92-1.07). There were more major haemorrhagic events among ASP+DP recipients (4.1%) than among clopidogrel recipients (3.6%) (HR 1.15; 95% CI 1.00 to 1.32). The net risk of recurrent stroke or major haemorrhagic event was similar in the two groups.

Serebruany et al. (2008) [26]: Their randomised single blind pilot study demonstrated that Asp+Dp was associated with a delayed but significant reduction in the expression of several activation-dependent platelet receptors, including glycoprotein (GP) IIb/IIIa (P = 0.02), platelet-endothelial cell adhesion molecule-1 (PECAM-1) (P = 0.03), GP Ib (P = 0.001), vitronectin (P = 0.001), P-selectin (P = 0.001), lysosome-associated membrane protein-1 (P = 0.001), and CD40 ligand (P = 0.01), as well as inhibition of both intact and cleaved epitopes of protease-activated receptor-1 by day 30 (P = 0.01). Conversely, clopidogrel monotherapy was associated with earlier and more potent antiplatelet activity, demonstrated by significant inhibition of adenosine diphosphate-induced platelet aggregation (P = 0.001), closure-time prolongation (P = 0.01), and reductions in rapid platelet function assay-ASA measurements by day 15 (P = 0.001). Clopidogrel treatment also significantly reduced PECAM-1 expression (P = 0.03) and GP IIb/IIIa activity (P = 0.01) by day 15. Clinically, these findings suggest that clopidogrel exerts more potent and earlier antiplatelet effects, whereas Asp+Dp produces broader but delayed downregulation of multiple platelet activation pathways.

Bath et al. (2010) [27]: Their single-blind study demonstrated that treatment with Asp+Dp vs clopidogrel in 1360 patients with acute, mild ischemic stroke 30 days after randomization, with adjustment for the covariates of age, sex, systolic blood pressure, severity, and antihypertensive assignment) did not differ between Asp+Dp and clopidogrel, analysed as an ordered categorical outcome (ordered modified Rankin Scale (mRS) categories 0, 1, 2, 3, and 4 to 6 to maintain proportionality) OR=0.97; 95% CI, 0.79 to 1.19; P=0.75) [27]. A trend to a reduction in stroke recurrence with Asp/ER-DP compared with clopidogrel was present at 90 days, whether assessed as time to event (OR=0.56; 95%, CI 0.26 to 1.18; P=0.12) or as a shift in the distribution of ordered categorical events (fatal stroke, dependent stroke (mRS 2 to 5), independent stroke (mRS 0, 1), transient ischemic attack, or no cerebrovascular event) (OR=0.75; 95% CI, 0.41 to 1.35; P=0.33).

Discussion

Main Findings

This structured literature review synthesized evidence from two large RCTs and one pilot study to compare the effectiveness of Asp+Dp with clopidogrel monotherapy for secondary prevention of ischemic stroke [12,26,27]. Across all included studies, there was no statistically significant difference between the two regimens in preventing recurrent stroke. In the largest trial, recurrent stroke occurred in 9.0% of Asp+Dp recipients and 8.8% of clopidogrel recipients over a mean follow-up of 2.5 years (HR 1.01; 95% CI 0.92-1.11) [12]. Functional outcomes, assessed at 30 days in one trial, were also similar between groups (OR 0.97; 95% CI 0.79-1.19; p = 0.29) [26]. Although clopidogrel produced faster platelet inhibition and Asp+Dp targeted additional activation pathways, these pharmacodynamic differences did not result in superior clinical outcomes within the follow-up periods [26]. Clinically, this suggests that variations in platelet inhibition profiles may not necessarily translate into meaningful differences in patient outcomes.

Comparison With Existing Literature

Earlier trials conducted prior to 2008, such as the MATCH trial (2004), suggested only modest differences in antiplatelet activity and failed to demonstrate clear clinical superiority of aspirin-based combinations over clopidogrel monotherapy. This present systematic review provides more robust evidence, consistently showing that aspirin plus dipyridamole and clopidogrel exhibit comparable efficacy in the secondary prevention of ischemic stroke [11,12]. These findings align with current guideline recommendations, including those from the American Heart Association/American Stroke Association, which endorse both regimens as appropriate first-line options for patients with non-cardioembolic ischemic stroke [9,28,29].

Strengths

This review has several strengths. It addressed a focused and clinically relevant research question framed by the PICO approach, ensuring clarity and precision. A comprehensive search strategy following PRISMA guidelines was employed to systematically identify eligible studies. To minimize bias, inclusion was restricted to randomized controlled trials and high-quality pilot studies, and methodological quality was rigorously assessed using the Cochrane Risk of Bias Tool.

Limitations

Only three studies met the inclusion criteria, restricting the ability to conduct subgroup analyses or a formal meta-analysis. Reported outcomes were largely short-term and underemphasized quality-of-life measures, limiting insights into long-term functional benefits. This wide range of follow-up durations (30 days to 2.5 years) complicates comparisons. The exclusion of non-English language publications may have resulted in the omission of relevant international evidence. Another limitation of this review is that quality-of-life (QoL) outcomes were only briefly addressed across the included studies, limiting the ability to draw meaningful conclusions about long-term patient-centered benefits. Also, another key limitation of this study is that only two databases were searched, which may have led to the omission of relevant studies and introduced selection bias. Moreover, two of the included studies were single-blind, which may have increased the risk of performance and detection bias, potentially affecting the reliability of their outcomes. Finally, adherence and real-world persistence with therapy were not systematically reported, leaving gaps in understanding practical treatment effectiveness.

## Conclusions

This structured literature review found no significant difference between aspirin plus extended-release dipyridamole and clopidogrel monotherapy in preventing recurrent ischemic stroke or improving short-term functional outcomes. Evidence from two large randomised controlled trials and one pilot study suggests that both regimens offer similar efficacy, with differences in pharmacodynamic profiles not translating into measurable clinical benefit during the study periods. Given these findings, either regimen can be considered an appropriate first-line option for the secondary prevention of non-cardioembolic ischemic stroke. Treatment choice should be guided by patient-specific factors. These include drug tolerability, comorbidities, dosing preferences, and cost considerations. Given the minimal number of included studies, further research is needed to examine long-term functional outcomes, adherence patterns, and potential benefits in specific patient subgroups. Clarifying these aspects could enable more tailored prescribing, improve patient outcomes, and optimise the use of healthcare resources, while acknowledging the limited generalizability of current evidence.
